# Региональные особенности основных показателей метаболизма углеводов у молодых жителей различных климатогеографических зон проживания

**DOI:** 10.14341/probl13582

**Published:** 2026-03-07

**Authors:** И. В. Аверьянова

**Affiliations:** Научно-исследовательский центр «Арктика» Дальневосточного отделения Российской академии наукРоссия; Scientific Research Center "Arktika" Far Eastern Branch of the Russian Academy of SciencesRussian Federation

**Keywords:** углеводный обмен, юноши, Север, Арктика, средняя полоса России, кортизол, гормонально-метаболический профиль, carbohydrate metabolism, young men, North, Arctic, central Russia, cortisol, hormonal and metabolic profile

## Abstract

**ОБОСНОВАНИЕ:**

ОБОСНОВАНИЕ. В различных регионах Российской Федерации колебания медико-биологических характеристик носят неоднозначный, а зачастую и разнонаправленный характер, при этом одним из путей к пониманию значимости действия данных компонентов является изучение их влияния на метаболическое состояние человека, в частности на углеводный обмен в идентичных по образу жизни группах, но проживающих в различных климатогеографических условиях.

**ЦЕЛЬ:**

ЦЕЛЬ. Оценка основных показателей углеводного обмена у молодых жителей различных климатогеографических зон проживания (северо-восток, северо-запад, средняя полоса России).

**МАТЕРИАЛЫ И МЕТОДЫ:**

МАТЕРИАЛЫ И МЕТОДЫ. Проведена оценка ключевых характеристик метаболизма углеводов у 243 лиц мужского пола, проживающих в различных регионах Российской Федерации: северо-восток России (г. Магадан) — 119 юношей, северо-запад России (г. Мурманск) — 72 юноши и средняя полоса России (г. Ульяновск) — 52 юноши. В работе использовали иммунохемилюминесцентный ферментативный метод и иммунохроматографический анализ.

**РЕЗУЛЬТАТЫ:**

РЕЗУЛЬТАТЫ. Установлено, что при сопоставимых рационах питания средние величины показателей метаболизма углеводов у обследуемых жителей различных регионов обследования соответствовали оптимальным физиологическим диапазонам с формированием региональных особенностей метаболического профиля. Выявлено смещение уровня гликемии и гликированного гемоглобина в сторону больших значений при низких концентрациях инсулина лишь в группе юношей северо-востока России на фоне повышенных значений сывороточного кортизола, наблюдаемых только в группах юношей-северян. Уроженцам северо-запада и средней полосы России была свойственна активация инсулярного аппарата поджелудочной железы, сопровождающаяся повышением уровня инсулина, высокой долей встречаемости признаков инсулинорезистентности (до 40% в выборке) при выраженной компенсаторной секрецией β-клеток поджелудочной железы.

Полученные данные свидетельствуют о том, что метаболические паттерны, наблюдаемые у северян, отличаются от классических критериев «полярного метаболического типа», которые обычно ассоциируются с гипогликемией, гипоинсулинемией на фоне повышенных значений сывороточного кортизола. При этом наличие признаков инсулинорезистентности в выборке юношей северо-запада России может быть обусловлено дисбалансом в сторону большего доминирования симпатического отдела вегетативной нервной системы.

**ЗАКЛЮЧЕНИЕ:**

ЗАКЛЮЧЕНИЕ. Полученные результаты свидетельствуют о том, что изменения в углеводном обмене имеют ярко выраженные «северные» особенности, но по-разному проявляющиеся у жителей северо-западных и северо-восточных регионов России. Установлено, что проживание в различных климатогеографических регионах Российской Федерации с их уникальными климатическими, географическими и широтными условиями приводит к формированию разнообразных вегетативных, эндокринных и метаболических профилей.

## Abstract (en)

**BACKGROUND:**

BACKGROUND: In different regions of the Russian Federation, fluctuations in biomedical characteristics are ambiguous and often multidirectional, while one of the ways to understand the significance of these components is to study their effect on the human metabolic state, in particular, on carbohydrate metabolism, in groups with identical lifestyles, but living in different climatogeographic conditions

**AIM:**

AIM: Assessment of the main indicators of carbohydrate metabolism in young residents of various climatic and geographical zones of residence (North-East, North-West, Central Russia).

**MATERIALS AND METHODS:**

MATERIALS AND METHODS: The assessment of the key characteristics of carbohydrate metabolism in 243 males living in various regions of the Russian Federation was carried out: 119 boys in the North-East of Russia (Magadan), 72 boys in the North-West of Russia (Murmansk) and 52 boys in the Middle of Russia (Ulyanovsk). The work used immunochemiluminescent, enzymatic method and immunochromatographic analysis.

**RESULTS:**

RESULTS: It was found that with comparable diets, the average values of carbohydrate metabolism in the surveyed residents of different regions corresponded to optimal physiological ranges with the formation of regional features of the metabolic profile. A shift in the level of glycemia and glycosylated hemoglobin towards high values at low insulin concentrations was revealed only in the group of young men in the North-East of Russia against the background of elevated serum cortisol values observed only in the groups of northern boys. Natives of Northwestern and central Russia were characterized by activation of the pancreatic insulin apparatus, accompanied by an increase in insulin levels, a high incidence of signs of insulin resistance (up to 40% in the sample), accompanied by pronounced compensatory secretion of pancreatic beta cells. The data obtained indicate that the metabolic patterns observed in Northerners differ from the classical criteria of the "polar metabolic type", which is usually associated with hypoglycemia and hypoinsulinemia on the background of elevated serum cortisol levels. At the same time, the presence of signs of insulin resistance in a sample of young men from the North-West of Russia may be due to an imbalance towards a greater dominance of the sympathetic division of the autonomic nervous system.

**CONCLUSION:**

CONCLUSION: The results obtained indicate that the identified changes in carbohydrate metabolism have pronounced «northern» features, but they manifest themselves differently in residents of the northwestern and northeastern regions of Russia. It has been established that living in various climatogeographic regions of the Russian Federation with their unique climatic, geographical and latitudinal conditions leads to the formation of diverse vegetative, endocrine and metabolic profiles.

## Обоснование

В различных регионах Российской Федерации (РФ) колебания медико-биологических характеристик носят неоднозначный, а зачастую и разнонаправленный характер, что определяется совокупностью средовых компонентов. Одним из путей к познанию и пониманию значимости действия данных компонентов является систематическое изучение их влияния на метаболическое состояние человека, в частности на углеводный обмен, изменение показателей которого отражает перестройку энергетических потоков при адаптации организма к холоду при этом наиболее гомеостатируемым метаболитом из числа изучаемых показателей обмена веществ у человека на севере является глюкоза [1][2]. Своеобразие региональных вариантов нормы в виде смещения эндокринных показателей относительно общепринятых среднеширотных норм, как правило, не слишком значительно, однако оно происходит разнонаправлено для различных гормонов, что приводит к формированию специфического «северного» гормонально-метаболического профиля организма человека — «полярного метаболического типа», при этом возникает совершенно иная структура внутри- и межгормональных связей [3][4][5][6][7]. Вопросы оптимальной адаптации и поддержания гомеостаза организма человека являются первостепенными применительно к различным по своим характеристикам, но при этом во многом одинаково суровым природно-климатическим условиям северо-востока и Заполярья северо-запада России. Несмотря на то, что северо-восток и Заполярье северо-запада России в принципе относятся к одному географическому понятию «Север», они в значительной степени отличаются по своим климатическим характеристикам, что не всегда можно объяснить различной широтностью данных регионов [8]. Актуальность обобщения современных представлений об адаптационных реакциях человека в условиях севера обусловливается повышенным вниманием к развитию арктической зоны РФ в связи с необходимостью защиты национальных интересов страны в этом стратегически важном регионе [9]. Понимание особенностей восприятия природно-климатических и антропогенных факторов риска различными социальными группами является необходимым условием построения эффективной политики в сфере здоровья населения, а также разработки успешной стратегии сохранения человеческого потенциала северных территорий [10].

## Цель исследования

Учитывая вышеизложенное, цель данной работы заключалась в оценке основных показателей углеводного обмена и его регуляции у молодых жителей различных климатогеографических зон проживания (северо-восток, северо-запад, средняя полоса России).

## Материалы и методы

## Место и время проведения исследования

Место проведения. В рамках проведения экспедиционных работ НИЦ «Арктика» ДВО РАН, направленных на оценку функциональных резервов жителей различных регионов РФ, в осенне-зимний период 2024 г. был проведен анализ ключевых показателей углеводного обмена у юношей из числа европеоидов 1–3 поколений, уроженцев и постоянно проживающих в различных климатогеографических зонах РФ: северо-восток (Дальний Восток, г. Магадан), северо-запад (арктическая зона РФ (АЗРФ), г. Мурманск) и средняя полоса России (умеренно-континентальная зона — г. Ульяновск). Исследования были проведены на базе Мурманского арктического университета и Ульяновского государственного университета. Полученные результаты сравнивались с аналогичными данными жителей северо-востока России (Северо-восточный государственный университет).

Время исследования. Исследование было проведено в осенне-зимний период 2024 г.

## Изучаемые популяции (одна или несколько)

В исследование были включены 3 популяции обследуемых представителей юношеского периода онтогенеза, проживающих в различных климатогеографических зонах РФ: северо-восток (Дальний Восток, г. Магадан), северо-запад (арктическая зона Российской Федерации (АЗРФ), г. Мурманск) и средняя полоса России (умеренно-континентальная зона — г. Ульяновск).

Критерии включения: мужской пол, юношеский период онтогенеза отсутствие хронических заболеваний в стадии обострения и жалоб на состояние здоровья, наличие информированного согласия. Все лица, вошедшие в выборку, характеризовались сопоставимыми условиями жизни (студенты) и режимом двигательной активности (занятия физической культурой в рамках плана образовательного учреждения) и являлись постоянными жителями исследуемого региона.

Критерии исключения: наличие любых острых либо декомпенсация хронических заболеваний на момент включения в исследование; отказ обследуемого от участия в исследовании.

## Способ формирования выборки из изучаемой популяции (или нескольких выборок из нескольких изучаемых популяций)

Выборка сформирована путем сплошного включения наблюдений с учетом критериев включения и исключения. Размер выборки предварительно не рассчитывался

## Дизайн исследования

Одномоментное сравнительное исследование.

## Описание медицинского вмешательства (для интервенционных исследований)

У испытуемых проводили взятие венозной крови натощак вакуумной системой в лаборатории ООО «Гемотест».

## Методы

Гликированный гемоглобин (HbA1с) определяли на автоматическом анализаторе D10 (Bio-Rad, США) с помощью референсного метода — жидкостной ионообменной хроматографии высокого давления. Инсулин определяли с использованием иммунохимического анализатора (IMMULITE 2000XPi, Siemens, США) на основе метода ферментативно-усиленной хемилюминесценции. Анализ в цельной венозной крови глюкозы натощак определяли гексокиназным методом на биохимическом анализаторе AU 680 (Beckman Coulter, США). Оценка инсулинорезистентности производилась на основе предложенной D.R Matthews и соавт. (1985) формуле для расчета индекс НОМА-IR:

[Инсулин (мкМе/мл) × Глюкоза (ммоль/л)]/22,5 [11].

Кортизол в сыворотке крови (нмоль/л) определяли методом ИХА с использованием автоматического иммунохемилюминесцентного анализатора Mindray CL 6000i.

Клинические диапазоны анализируемых показателей в соответствии с протоколом медицинской лаборатории «Гемотест» имели следующие размахи: кортизол (утро) — 171,05–535,21 нмоль/л, гликированный гемоглобин — до 6% включительно, глюкоза натощак — 4,11–6,1 ммоль/л, индекс инсулинорезистентности (HOMA-IR) менее 2,7 усл. ед., инсулин — 2,6–24,9 мМе/мл.

Из полученных показателей производили расчет индекса инсулин/кортизол, оптимальное значение которого оценивали по референсной медиане референсных диапазонов для инсулина и кортизола равной 2,5 усл. ед. [8].

Расчет НОМА-β, отражающий функциональную (секреторную) способность β-клеток поджелудочной железы, проводили по следующей формуле:

[ 20 × IRI Инсулин (мкМе/мл)] / [ Глюкоза (ммоль/л) — 3,5] [12].

Критерием нарушения углеводного обмена (нарушение гликемии натощак) согласно ADA, 2019, считали уровень глюкозы равный 5,6–6,9 ммоль/л [13]. Выявление инсулинорезистентности проводили с помощью метода оценки гомеостатической модели (HOMA-IR) с точкой отсечения >2,27 усл. ед. [14]. За нормальную секреторную функцию β-клеток принимался показатель HOMA-β, равный 100% [15]. Гиперинсулинемия диагностировалась при уровне инсулина >12,09 мМе/мл [16]. Повышенные концентрации кортизола регистрировались при значениях, превышающих 314 нмоль/л — верхнюю границу физиологической нормы, установленную для здоровой популяции северных регионов [17].

Скорость потребления кислорода VО2 (мл/мин) определяли с помощью мeтаболографа Спиролан-М (Ланамедика, Россия).

Вариабельность кардиоритма регистрировалась при помощи комплекса «Варикард» и программного обеспечения VARICARD-KARDi. В работе использовался индекса LF/HF, усл. ед., отражающий соотношение мощности низкочастотных (LF, мс²) и высокочастотных колебаний (HF, мс²).

Оценку рациона и типа питания осуществляли путем фиксирования суточного дневника питания и анализа суточных рационов питания с использованием программы «АСПОН-питание» (г. Санкт-Петербург) с вычислением среднего значения макронутриентного состава рациона питания и его суточного калоража. С использованием данной программы были проанализированы следующие компоненты суточного рациона: белки (г), жиры (из которых жиры растительного и животного происхождения) (г), углеводы (с выделением моно- и дисахаридов, а также поведена оценка энергетической ценности суточного рациона питания (ккал/сут.).

## Статистический анализ

Статистическую обработку полученных данных проводили с использованием программы IBM SPSS Statistics 21.0. Проверка на нормальность распределения измеренных переменных осуществлялась на основе теста Шапиро-Уилка. Результаты непараметрических методов обработки представлены в виде медианы (Me) и интерквартильного размаха в виде 25 и 75 процентилей, а параметрических, как среднее значение и его ошибка (М±m). При множественном сравнении для выборок с нормальным распределением был использован параметрический однофакторный дисперсионный анализ (ANOVA). Далее, для выявления статистически значимых различий между конкретными группами нами был использован апостериорный анализ с помощью теста для множественных сравнений Scheffe. При множественном сравнении выборок с распределением, отличающимся от нормального, был использован ранговый дисперсионный анализ Краскела-Уоллиса. Также по всем показателям вычислялись отклонения в % от физиологической нормы или общепринятых референтных значений, в соответствии с пределами, указанными выше. Критический уровень значимости (p) в работе принимался равным 0.05; 0.01; 0.001.

## Этическая экспертиза

Исследование было выполнено в соответствии с принципами Хельсинской декларации (2013 г.). Протокол исследования был одобрен Локальным этическим комитетом Федерального государственного бюджетного учреждения науки Научно-исследовательский центр «Арктика» Дальневосточного отделения Российской академии наук (заключение № 002/021 от 26.11.2021 г.). До включения в исследование у всех участников было получено письменное информированное согласие.

## Результаты

В исследование были включены 243 представителя мужского пола из которых 72 обследуемых юношеского возраста (19,4±0,2 года) с индексом массы тела (ИМТ) 23,1±0,4 кг/м², проживающих в АЗРФ (г. Мурманск). В г. Ульяновске (средняя полоса России) в исследование были включены 52 юноши, средний возраст и ИМТ которых составил 19,9±0,3 года и 23,1±0,5 кг/м² соответственно. В северо-восточном регионе (г. Магадан) были обследованы 119 юношей (19,5±0,2 года) с ИМТ, равным 23,0±0,4 кг/м².

Анализ показателей углеводного обмена, результаты которого представлены в таблице 1, свидетельствует о значимых перестройках, проявляющихся в региональных особенностях метаболизма углеводов в зависимости от локации проживания. Стоить отметить, что оценка суточной калорийности и химического состава рационов питания обследуемых трех групп (табл. 2) свидетельствует об их сопоставимости с выраженный липидной направленностью и достаточной долей монодисахаридов в рационе питания, достигающей 12–15% от суточной калорийности (15% — юноши северо-запада, 14% — северо-восток России, 12% — юноши средней полосы России).

**Table:** Таблица 1. Показатели углеводного обмена, кортизола, соотношения LF/HF вариабельности сердечного ритма у юношей — жителей различных климатогеографических зон Российской Федерации Примечание. HbA1с — гликированный гемоглобин, %; HOMA-IR — индекс инсулинорезистентности, усл. ед.; НОМА-β — индекс секреторной активности β-клеток поджелудочной железы; LF/HF — индекс соотношения низкочастотных и высокочастотных колебаний, отражающий симпато-вагальный баланс.

Изучаемые показатели	Локация научно-исследовательских работ	Уровень значимости различий
Северо-запад РФ (1)	Северо-восток РФ (2)	Средняя полоса РФ (3)
1–2	2–3	1–3
Глюкоза, ммоль/л	4,64±0,07	4,99±0,06	4,80±0,04	p<0,05	p<0,05	p<0,05
Инсулин, мМе/мл	10,43±0,61	7,74±0,34	10,12±0,79	p<0,001	p<0,001	p=0,75
HbA1с, %	4,95±0,02	5,18±0,02	4,86±0,03	p<0,001	p<0,001	p<0,05
HOMA-IR, усл. ед.	2,18±0,13	1,78±0,08	2,19±0,18	p<0,001	p<0,01	p=0,96
НОМА-β, усл. ед.	183,5±8,2	103,9±6,2	155,7±4,5	p<0,001	p<0,001	p<0,01
Кортизол, нмоль/л	401,3±13,19	426,7±18,1	350,40±18,0	p=0,26	p<0,01	p<0,01
LF/HF, усл. ед.	2,8 (1,2; 4,4)	1,5 (0,9; 2,8)	2,1 (1,3; 3,1)	p<0,01	p<0,05	p<0,05

**Table:** Таблица 2. Макронутриентный состав рациона у юношей — жителей различных климатогеографических зон Российской Федерации

Изучаемые показатели	Локация научно-исследовательских работ
Северо-запад РФ	Северо-восток РФ	Средняя полоса РФ
Белки, г	85,4±5,4	97,6±5,6	103,6±6,2
Жиры, г	105,2±5,9	101,4±5,9	103,0±6,2
Углеводы, г	185,9±13,4	213,8±11,7	176,9±8,0
Монодисахариды, г	75,5±6,5	77,5±6,3	61,3±6,6
Энергетическая ценность, ккал	2072,6±95,6	2239,7±75,6	2107,0±92,9

Концентрация глюкозы в крови оказалась на статистически значимую величину ниже в выборке молодых жителей Заполярья сравнительно с юношами средней полосы, в выборке которых данный показатель был значимо ниже, чем у юношей северо-востока России. Показано, что группе молодых людей — уроженцев северо-запада России были характерны значимо более низкие показатели глюкозы и гликированного гемоглобина с отсутствием признаков гипоинсулинемии, тогда как значимо более низкие показатели базального инсулина на фоне самых высоких межгрупповых значений глюкозы натощак были зафиксированы в группе уроженцев северо-восточного региона России.

Как видно из представленной таблицы, концентрация НbА1с в крови у юношей трех групп соответствовала референсным величинам, однако была на значимую величину выше у уроженцев северо-востока России, тогда как самые низкие числовые значения были зафиксированы в выборке средней полосы. Показано, что индекс HOMA-IR как маркер степени инсулинорезистентности вплотную приближался к верхней границе оптимального диапазона в выборках юношей северо-запада и средней полосы России с достаточно высокой долей встречаемости лиц с признаками инсулинорезистентности (в 39 и 38% случаев соответственно), что наблюдалось на фоне высоких значений HOMA-β, отражающих компенсаторное возрастание секреторной функции β-клеток поджелудочной железы. Статистически значимо более низкие показатели HOMA-β, соответствующие оптимальному физиологическому диапазону были характерны для юношей северо-востока России.

В группе юношей-северян в сравнении со сверстниками из средней полосы России были отмечены значимо более высокие средние концентрации сывороточного кортизола с тенденцией к превалированию в группе молодых людей северо-востока России.

Оценку вегетативного контроля сердечно-сосудистой системы проводили по показателю LF/HF, величина которого в группе юношей средней полосы составила 2,1 (1,3; 3,1) усл. ед. и свидетельствовала о равноценном вкладе как парасимпатической, так и симпатической активности в регуляции (нормотония). В выборке уроженцев северо-запада России медианное значение LF/HF равнялось 2,8 (1,2; 4,4) усл. ед., что указывает на симпатическое доминирование в вегетативной регуляции, тогда как в группе юношей северо-востока показатель симпатовагального баланса составил 1,5 (0,9; 2,8) усл. ед., величина которого отражает сдвиг в область парасимпатического преобладания в регуляции кардиоритма.

## Обсуждение

## Репрезентативность выборок

Проведенные экспедиционные исследования позволили установить, что у всех обследуемых юношей средние величины ключевых характеристик углеводного обмена находились в диапазоне референсных значений, но вместе с тем были обнаружены значимые межгрупповые отличия, обусловленные регионом проживания.

## Сопоставление с другими публикациями

Известно, что поддержание необходимого уровня глюкозы в крови находится под контролем сложного «ансамбля» регуляторных систем, которые обеспечивают координацию процессов ее мобилизации и утилизации [18]. Накопленные к настоящему времени данные свидетельствуют, что у жителей-северян в основе перестройки энергетического обмена лежит изменение гормональной регуляции: некоторое увеличение концентрации глюкокортикоидов на фоне выраженного снижения содержания инсулина в крови [19][20]. Учитывая вышеописанный контринсулярный механизм взаимодействия кортизола и инсулина, широко освещенный в работах по изучению эндокринно-метаболических перестроек жителей-северян, был проведен анализ концентрации кортизола в крови у обследуемых групп юношей. Проведенные исследования позволили выявить «северные» особенности функциональной активности гипоталамо-гипофизарно-надпочечниковой оси, проявляющиеся в высоких средних концентрациях сывороточного кортизола в выборках юношей-северян, с тенденцией к возрастанию данных величин в группе молодых людей северо-востока России.

Показано, что значимо низкие показатели базального инсулина, сочетаемые с превалирующими значениями глюкозы натощак и сывороточного кортизола, были зафиксированы в группе уроженцев северо-восточного региона России, что может быть проявлением ведущего механизма мобилизации энергетических ресурсов в условиях севера, проявляющегося переключение «углеводного» типа метаболизма на «белково-липидный», при котором отмечаются аналогичные перестройки вышеуказанных гормонов [15][16]. Полученные данные указывают на то, что в выборке юношей-магаданцев достаточно высокие значения концентрации кортизола в крови оказывали контргормональное влияние на концентрацию инсулина в виде низких значений базального инсулина, но в пределах референсного диапазона, на фоне более высоких показателей глюкозы натощак, что соответствует концепции действия высоких концентраций контрискулярных гормонов в формировании относительной «северной инсулярной недостаточности» и «диабета напряжения» при проживании на севере [19][20]. Представлены факты, что уменьшение доступности инсулина или избыток его антагонистов (кортизола) активирует ключевые ферменты липолиза, при котором периферические ткани организма в большей степени обеспечиваются энергией за счет утилизации липидных субстратов [19][20]. Накопленные к настоящему времени данные свидетельствуют, что при возрастании доли использования жиров в качестве энергетического субстрата увеличивается потребление кислорода [21]. Это согласуется с результатами нашего исследования, в которых скорость потребления кислорода в выборке юношей северо-востока России (292,2±4,68 мл/мин) была на значимую величину выше, чем у сверстников из Заполярья (262,5±9,63 мл/мин (p<0,01)) и в выборке жителей средней полосы России (272,6±10,6 (p<0,05)). Высокие значения скорости потребления кислорода у юношей северо-восточного региона свидетельствуют об активизации аэробного окисления веществ, при котором для поддержания энергетического гомеостаза увеличивается роль окисления жирных кислот наблюдаемое на фоне значимо более высоких величин глюкозы и гликированного гемоглобина. О повышении роли липидов в энергообеспечении адаптационных процессов свидетельствует и более низкое соотношение инсулин/кортизол в крови, величина которого у магаданцев составила 1,81 усл. ед., у мурманчан — 2,59 усл. ед., у юношей средней полосы — 2,88 усл. ед.

Как показывают результаты исследования, для уроженцев северо-западного региона были характерны значимо более низкие показатели глюкозы и гликированного гемоглобина, соответствующие критериям «полярного метаболического типа», но с отсутствием характерной для данного типа гипоисулинемии, наблюдаемой при высоких значениях концентрации кортизола. Вывяленные в выборке юношей Заполярья сниженные величины скорости потребления кислорода, сочетаемые с низкими значениями концентрации глюкозы и гликированного гемоглобина в крови, могут являться отражением меньшей интенсивности аэробного окисления веществ и усиления роли анаэробного гликолиза в обеспечении энергетических потребностей организма. Примечательно, что в группе уроженцев северо-запада России контргормонального взаимодействия в системе «кортизол-инсулин» зафиксировано не было на фоне проявления низких величин глюкозы натощак, наблюдаемые при высокой доле проявления инсулинорезистентности (у 38% юношей, против 13% в выборке магаданцев). По мнению авторов, инсулинорезистентность может являться приспособительной реакцией, предназначенной для усиления и перераспределения потоков энергетических субстратов в клетки под действием влияния экстремальных факторов окружающей среды и носит физиологический (адаптивный) характер [22]. При этом указывается, что в основе нарушений метаболизма углеводов, проявляющихся наличием признаком инсулинорезистентности лежит неадекватная и/или избыточная продукции глюкокортикоидных гормонов и активации гипоталамо-гипофизарной системы (ГГС) [22]. Необходимо отметить, что ГГА является основным медиатором реакции на стресс и функционирует в тандеме с другим важнейшим медиатором, таким как вегетативная нервная система (ВНС), в частости ее симпатическое звено, которые активируются параллельно [23]. Кроме того, показано, что вагусный тонус играет ключевую роль в регуляции ряда аллостатических систем, включая сердечно-сосудистую систему, регуляцию уровня глюкозы, функцию гипоталамо-гипофизарно-надпочечниковой системы [24][25]. В настоящее время анализ вариабельности сердечного ритма широко используется для оценки состояния и гибкости (адаптивности) вегетативной нервной системы, однако данный подход используется не только как маркер регуляции сердечно-сосудистой функции, но и как показатель общего физического благополучия, как индикатор нарушений в гипоталамо-гипофизарно-надпочечниковой системе [26], как предиктор заболеваемости и смертности [27]. Накопленные к настоящему времени данные свидетельствуют об ассоциации между активностью вегетативной нервной системы, оцененной по показателям вариабельности сердечного ритма и концентрацией в моче биомаркеров симпатической активности — норадреналина и адреналина [28].

С учетом вышеизложенного отдельный интерес представлял анализ вегетативного обеспечения метаболизма углеводов, учитывая их региональных особенностей.

Анализ характеристик вариабельности сердечного ритма указывает на то, что в популяции молодых жителей проживание в условиях Заполярья приводит к снижению вегетативных функций за счет уменьшения активности парасимпатического звена вегетативной нервной системы, смещающего симпатовагальный баланс в относительное состояние симпатической активности, тогда как молодые жители северо-восточного региона России демонстрировали доминирование активности парасимпатического звена вегетативной нервной системы. В выборке юношей средней полосы России был зафиксирован равный вклад парасимпатического и симпатического звеньев в обеспечение вегетативной регуляции (нормотония).

Важно отметить, что выявленное нами снижение модуляции парасимпатического на фоне активации симпатического звена в вегетативной регуляции у юношей Заполярья совпадало с весьма значительным процентом лиц с признаками инсулинорезистентности. Известно, что симпатическая иннервация имеет решающее значение для регуляции выделения инсулина и глюкагона поджелудочной железой и контроля гомеостаза глюкозы, при этом доминирование симпатического отела вегетативной регуляции приводит к снижению чувствительности инсулиновых рецепторов [29]. Как нам представляется, сдвиг в область преобладания симпатической активности в вегетативной регуляции может обусловливать выявленные в нашем исследования признаки инсулинорезистентности в выборке юношей северо-запада России.

Характер полученных изменений показателей в наших исследованиях свидетельствует о том, что снижение концентрации инсулина на фоне самых высоких величин глюкозы у юношей северо-востока наблюдались в сочетании с выраженным доминированием парасимпатического звена в вегетативной регуляции. В последнее время в литературе указывается на связь активности поджелудочной железы с нейромедиатором вагусной активности — ацетилхолином. Показано, что поджелудочная железа активируется более низкими концентрациями ацетилхолина, чем надпочечники, а дальнейшее повышение его уровня приводит к угнетению секреции данной железы и активации надпочечников, т.е. действие ацетилхолина на секрецию инсулина поджелудочной железой имеет дозозависимый эффект, при которой повышение концентрации ацетилхолина до уровня, возбуждающего секрецию надпочечников, угнетает секрецию инсулина [30].

Полученные в наших исследованиях результаты согласуются с литературными данными и могут быть объяснением трансформации углеводного обмена в виде низких уровней базального инсулина на фоне значимо более высоких величин глюкозы в сочетании с вагусно-опосредованной вариабельностью сердечного ритма в группе юношей северо-востока России.

Стоить отметить, что основные показатели метаболизма углеводов уроженцев средней полосы России были полностью сопоставимы с аналогичными данными представителей северо-запада, проявляющиеся высокой частотой встречаемости признаков инсулинорезистентности (39%), активацией секреторной функции β-клеток поджелудочной железы, но которые наблюдались на фоне оптимальных величин концентрации сывороточного кортизола и нормотонии в вегетативной регуляции.

## Клиническая значимость результатов

Полученные результаты свидетельствуют о том, что изменения в углеводном обмене имеют ярко выраженные «северные» особенности, но по-разному проявляющиеся у жителей северо-западных и северо-восточных регионов России. Установлено, что проживание в различных климатогеографических регионах Российской Федерации с их уникальными климатическими, географическими и широтными условиями приводит к формированию разнообразных вегетативных, эндокринных и метаболических профилей.

## Ограничения исследования

Ограничением нашего исследования является недостаточно широкий охват населения регионов РФ. Наше исследование имеет некоторые ограничения, основным является участие в исследовании лишь лиц мужского пола, что не позволяет в полной мере описать популяцию жителей-северян. Также применение наших результатов может быть ограничено только европеоидной этнической принадлежностью.

## Направления дальнейших исследований

Проведение аналогичного анализа в выборке зрелого возраста.

## Заключение

Проанализированы механизмы регуляции углеводного обмена, проявляющиеся в ряде различий в зависимости от региона проживания. Полученные результаты свидетельствуют о том, что формирование перестроек углеводного обмена, несомненно, имеет свою «северную» специфику, но с проявлением различных метаболических профилей у жителей северо-запада и северо-востока России. Проведенные исследования позволили установить, что проживание в условиях северных территорий, различающихся по климатогеографическому расположению, а также широтным характеристикам, обусловливает формирование различных вегетативно-эндокринно-метаболических профилей.

По-видимому, у уроженцев северо-востока России, в отличие от сверстников северо-запада России и средней полосы проживания, происходит перестройка энергетического обмена, которая сопровождается повышением роли липидов в энергообеспечении адаптационных реакций, что подтверждается увеличением потребления кислорода, характерным представителям этой конкретной группы, ассоциированным с высокими значениями соотношения кортизол/инсулин и доминированием парасимпатического звена в вегетативной регуляции.

В группе уроженцев северо-западного региона России значимо более низкие показатели глюкозы и гликированного гемоглобина соответствовали критериям «полярного метаболического типа», но при отсутствии контргормонального взаимодействия в системе «кортизол-инсулин», что проявлялось отсутствием низких значений базального инсулина при высоких концентрациях кортизола. Довольно высокая частота признаков инсулинорезистентности у представителей данной выборки, по-видимому, обусловлена доминированием симпатического звена вегетативной регуляции. Эти перестройки в углеводном обмене сопровождались низким потреблением кислорода, что, по-видимому, является отражением меньшей интенсивности аэробного окисления веществ и повышенной роли анаэробного гликолиза в обеспечении энергетических потребностей организма.

Наличие маркеров, свидетельствующих об инсулинорезистентности, в сочетании с активацией секреторной активности β-клеток поджелудочной железы, в то время как остальные параметры углеводного обмена находились в пределах диапазона физиологических значений, на фоне оптимального баланса моно- и дисахаридов в рационе и физиологической концентрации сывороточного кортизола в выборке уроженцев средней полосы России, несомненно, требует дальнейшего изучения.

Обнаружена ассоциация между регионом проживания и соотношением гормонов инсулин/кортизол крови у исследуемых групп, величина которого была специфична и возрастала в ряду: северо-восток — северо-запад – средняя полоса России. Изучены особенности адаптивных перестроек в системе метаболизма углеводов во взаимодействии с активностью ГГО-оси и вегетативной регуляции у жителей двух северных регионов сравнительно с регионом проживания, характеризующимися условно-оптимальными климатическими условиями.

Полученные данные демонстрируют существование четко выраженных регион-специфических адаптационных механизмов, сформировавшихся под влиянием длительного воздействия различных климатогеографических факторов. Несмотря на тот факт, что выявленные физиологические особенности углеводного обмена у исследуемых выборок находятся в пределах общепринятых референтных диапазонов, их специфический характер может создавать предрасположенность к развитию определенных патологических состояний, характерных для конкретных географических популяций. Полученные в нашем исследовании выводы подчеркивают важность разработки дифференцированных подходов к профилактическим мероприятиям, учитывающим не только индивидуальные метаболические профили, но и региональные особенности их физиологической адаптации. Такой персонализированный подход представляется особенно актуальным для жителей экстремальных климатических зон, где адаптационные механизмы подвергаются наибольшему напряжению.

## ДОПОЛНИТЕЛЬНАЯ ИНФОРМАЦИЯ

Источники финансирования. Работа выполнена за счет бюджетного финансирования НИЦ «Арктика» ДВО РАН в рамках выполнения темы «Комплексные молекулярно-генетичнские и морфофункциональные детерминанты адаптационных перестроек в условиях Севера» (рег. номер 1025031800012-3-3.1.8)

Конфликт интересов. Автор декларирует отсутствие явных и потенциальных конфликтов интересов, связанных с публикацией настоящей статьи.

Участие авторов. Автор подтверждает соответствие своего авторства международным критериям ICMJE (автор внес существенный вклад в разработку концепции, проведение исследования и подготовку статьи, прочли и одобрили финальную версию перед публикацией). Вклад распределен следующим образом: Аверьянова И.В. — разработка концепции и дизайна исследования, организация экспедиционных исследований в г. Мурманске и г. Ульяновске, организация взятия крови для оценки биохимических показателей в сторонней организации (ООО «Гемотест»), формирование базы данных, анализ и интерпретация данных, обзор литературных источников, написание текста и редактирование статьи.

Благодарности. Автор рукописи выражает благодарность Балыкину Михаилу Васильевичу, Мишаниной Людмиле Александровне за помощь в организации обследуемых контингентов.
